# Level of evidence in high impact surgical literature: the way forward

**DOI:** 10.1007/s13304-024-01961-w

**Published:** 2024-08-11

**Authors:** Hassan ElHawary, Joseph Kaleeny, Omar ElSewify, Barbara Granicz, Sukhmeet Singh Sachal, Victor Kang, Jad Abi-Rafeh, Jeffrey E. Janis

**Affiliations:** 1 Division of Plastic and Reconstructive Surgery, McGill University Health Centre, Montreal, QC Canada; 2 Department of Plastic and Reconstructive Surgery, Wexner Medical Center, The Ohio State University, 915 Olentangy River Rd., Columbus, OH 43210 USA; 3 Faculty of Medicine and Health Sciences, Laval University, Quebec, Canada; 4 Indiana University School of Medicine, Indianapolis, USA; 5 Division of Plastic and Reconstructive Surgery, Western University, London, Canada; 6 Faculty of Medicine, McGill University, Montreal, QC Canada

**Keywords:** Bibliometric analysis, Level of evidence

## Abstract

Evidence-based medicine stipulates that clinical decision-making should revolve around scientific evidence. The goal of the present study is to evaluate the methodological quality of surgical research recently published in JAMA Surgery, International Journal of Surgery, and British Journal of Surgery, the three surgical journals with the highest impact factor. An electronic search of the PUBMED database was performed to retrieve all articles published in the JAMA Surgery, International Journal of Surgery, and British Journal of Surgery in the year 2022. Three authors independently reviewed all retrieved articles and methodological designs of the publications were analyzed and rated using a modification of the Oxford Centre for Evidence-Based Medicine Levels of Evidence (Oxford Levels of Evidence scale). The initial search identified 1236 articles of which 809 were excluded after title and abstract screening. The remaining 427 underwent full text/methods read, of which 164 did not meet the inclusion/exclusion criteria. A total of 273 studies were included in the analysis. The average level of evidence was 2.5 ± 0.8 across all studies assessed. The majority of study designs were comprised of retrospective cohorts (*n* = 119), prospective cohorts (*n* = 47), systematic reviews of non RCTs (*n* = 39), and RCTs (*n* = 37). There was no significant difference in the average level of evidence between the top three journals (*p* = 0.50). Most clinical studies in the highest impact factor surgical journals are of level III evidence, consistent with earlier literature. However, our analysis demonstrates a relatively higher percentage of LOE I and II compared to what was previously published in the literature.

## Introduction

Medical literature has experienced rapid growth in recent years [1, 2]. Surgical practice relies on a rigorous investigation of the scientific literature as the field continues to evolve [1–3]. Prior research on study design strength has raised concerns over the stagnancy of high-quality surgical publications, which ultimately means that surgeons may have to rely on lower levels of published evidence to draw scientific conclusions that can impact practice [4–7].

Classifying studies by their level of evidence (LOE) provides a means to assess their methodological design. Prospective, randomized controlled trials remain the gold standard of clinical research. Multiple studies have demonstrated the scarcity of high level of evidence publications in the surgical field [8–11]. However, it remains unclear whether this is true for the highest impact factor surgical journals.

The goal of this study was to evaluate the methodological quality of surgical research published in 2022 in the top three journals with the highest impact factor: JAMA Surgery (JAMA Surg), International Journal of Surgery (Int Journal of Surg), and British Journal of Surgery (BJS). Moreover, this study serves as a call to action for surgeon scientists and surgical institutions to implement strategies that encourage improving LOE and the quality of surgical research.

## Materials and methods/literature search

An electronic search of the PUBMED database was performed to retrieve all articles published in JAMA Surg, Int Journal of Surg, and BJS from January 1st to December 31st of 2022. No IRB approval or consent was needed for this study. Three authors (JK, OS, and BG) independently reviewed all articles. All discrepancies between the three authors were reviewed and resolved by the primary author (HE). Inclusion criteria were any clinical study published in these journals in the year 2022. Nonclinical studies such as animal models, laboratory experiments, surgical techniques, editorials, letters to the editor, reviews, abstracts, or miscellaneous articles were excluded from the study. The papers related to COVID-19 were also excluded.

Each paper included was classified based on the study’s methodology into one of the following categories: (1) randomized control trials, (2) systematic review and meta-analysis of randomized controlled trials, (3) prospective cohort studies, (4) retrospective cohort studies, (5) case control studies, (6) cross-sectional studies, (7) case series, (8) case reports, and (9) reviews and/or meta-analysis of studies other than randomized controlled trials. The level of evidence of articles was determined using a modification of the Oxford Centre for Evidence-Based Medicine Levels of Evidence (Oxford Levels of Evidence scale), utilized in previous similar studies [12]. The level of evidence of systematic reviews and/or meta-analysis was determined based on the lowest level of evidence of the primary studies included in the review (i.e., systematic review and/or meta-analysis of retrospective cohort studies was determined to be a level of evidence 3).

### Statistical analysis

The weighted level of evidence of each journal was calculated by adding all the levels of evidence of the individual articles divided by the number of articles. Chi-square analyses were performed to analyze for significant differences between categorical outcomes while analysis of variance (ANOVAs) was used to measure significant differences between continuous variables. A pre-determined *p*-value of < 0.05 was considered statistically significant.

## Results

The initial search on PUBMED identified 1236 papers published in JAMA Surg (388), IJS (476), and BJS (372), of which 809 were excluded after title and abstract screening. The remaining 427 underwent full text/methods read, of which 164 failed inclusion/exclusion criteria. The final analysis was done on 273 clinical studies. Most of the publications were related to general surgery (*n* = 113; 41.4%), followed by oncology (*n* = 77; 28.2%), vascular (*n* = 14; 5.1%), and plastic surgery (*n* = 11; 4.0%).

The most common type of study published was retrospective cohort (*n* = 119; 43.6%) followed by prospective cohort, (*n* = 47; 17.2%), systematic reviews of non-RCTs (*n* = 39, 14.3%), and RCTs (*n* = 37; 13/6%). There was a total of 11 systematic reviews of RCTs (4%), 10 case series/reports (3.7%), 6 cross-sectional studies (2.2%), and 4 case control studies (1.5%). (Table 1). Approximately two-thirds of all included studies were level III evidence (*n* = 164; 60%). A total of 48 studies (17.6%) and 47 studies (17.2%) were level I and II evidence, respectively. Very few studies were LOE IV (*n* = 10; 3.7%). The average level of evidence was 2.5 ± 0.8. There were no significant differences in LOE between the journals (JAMA: 2.43, Journal of Int Surg: 2.55, BJS: 2.56 (*f* = 0.69; *p* = 0.5). Systematic reviews of non-RCTs were published significantly more in the Journal of Int Surg compared to both JAMA Surg and BJS (*p* < 0.001). There were no differences in the proportion of various study types published between the journals otherwise (Table 1).

**Table 1:** Distribution of different types of study and their level of evidence across JAMA

Type of study	Level of evidence	JAMA Surg	International Journal of Surgery	British Journal of Surgery	Total	*P* value
RCT	1	17	7	13	37	0.29
SR and/or MA of RCT	1	2	6	3	11	0.14
Prospective cohort	2	20	9	18	47	0.28
Retrospective cohort	3	51	30	38	119	0.14
Case control	3	3	1	0	4	N/A
Cross sectional	3	3	1	2	6	0.83
Case series	4	1	1	0	2	N/A
Case report	4	0	0	8	8	N/A
Review of non RCT^a^	Mixed	2	23	14	39	< 0.001
Level of Evidence	–	2.43 + 0.82	2.55 + 0.78	2.56 + 0.88	2.51 + 0.83	0.50
Total		99	78	96	273	

Surgery, International Journal of Surgery, and British Journal of Surgery

^a^These studies have varying levels of evidence based on the level of evidence of the primary studies that were included in the reviews

## Discussion

The current analysis shows that the majority of clinical studies published in the three highest impact factor surgical journals consist of low level of evidence (III–IV). The average level of evidence in all three journals in year 2022 was 2.5.

A review of the literature reveals that most previous surgical research is categorized as level III evidence, aligning with the results of our analysis [13–15]. However, results from the current study show that level I and II studies were more prevalent and level IV was less prevalent in the top three highest impact factor journals compared to previously published surgical literature [13–16]. For example, a recent article assessed the level of evidence in three major thoracic surgery journals and showed that only 1.4% and 3.7% of the studies were levels I and II, respectively (vs 17.6% and 17.2% in the current study) [8]. A similar trend is observed in many other surgical subspecialties such as orthopedics [17], plastic surgery [18], otolaryngology [19].

There are multiple possible reasons for the overall low level of evidence in the surgical domain. Firstly, randomizing surgical procedures is challenging since most surgeons are trained to preform surgeries in a specific manner and novel surgical techniques are not as common as novel medical treatments. For example, it would be logistically more feasible to randomize diabetic patients to receive two different types of insulin compared to randomizing cancer patients to receive two different surgical resection approaches. Another possible culprit of the low level of evidence demonstrated in surgery is the paucity of full-time surgeon researchers. While there are surgeons who exclusively conduct research, the majority of surgeons involved in research split their time between their clinical and scientific duties. Moreover, conducting an RCT by definition requires the investigators to be in a state of equipoise with regards to which treatment is better. Many surgeons would not be comfortable consciously knowing they are uncertain of the efficacy of the surgery they are performing [20]. Finally, the lack of funding and scientific infrastructure in surgical research is a significant obstacle to conducting higher level of evidence research, which is usually costly and requires robust infrastructure. Dedicated full time researchers are more likely to receive research grants that allows them to conduct randomized trials or prospective cohort studies. One of the factors that funding bodies consider is the previous quality of research published, which can lead to a viscous cycle where low quality of research leads to less funding which further fuels low level quality research.

An important distinction to be emphasized is that the level of evidence does not always directly equate with impact of research, nor does funding [21]. The level of evidence does not include crucial factors such as sample size, blinding techniques, and clinical applicability of results. An underpowered unblinded randomized controlled trial (LOE I) can provide minimal information to help guide clinical decisions. Conversely, a large well conducted retrospective comparative cohort trial (LOE III) with well-conducted statistical analysis that controls for cofounding factors can provide important information that guides clinical decision making. Therefore, it is important to perceive level of evidence as one way of assessing research methodology, but remember that other important factors need to be considered as well.

As surgeons, it is our responsibility to enhance clinical management guided by rigorous scientific evidence. Drawing from the literature and the author's practical experience, the following are suggested tactics to improve LOE and the quality of surgical research:

Clear research questions and hypotheses need to be established prior to conducting the study may allow investigators to choose the most appropriate study design.

1. Research questions and hypotheses need to be very clear prior to conducting the study in order to allow the investigators to choose the highest level of evidence study design possible.
2. Hiring a research coordinator can help streamline research protocols, follow up with patients longitudinally and facilitate larger prospective studies.
3. Collaboration between different surgical centers can help increase sample size especially when studying rare pathologies/cases [22].
4. Thorough discussions with expert statisticians prior to conducting the study can help identify the best analysis possible for a specific study design.
5. Institutional incentives should be implemented to surgeons conducting research and continued educational resources should be provided to interested surgeons to keep them up to date with epidemiology/statistics.

## Conclusion

The current study demonstrates that the majority of clinical studies published in the highest impact factor surgical journals consist of level III evidence, in line with earlier literature. Encouragingly, our analysis demonstrates a relatively higher percentage of LOE I and II and a lower percentage of LOE IV compared to other surgical journals. We hope that this paper serves as a call to action for surgeon-scientists and surgical institutions to implement strategies to propel the surgical research field forward.

## Data Availability

Not applicable.
